# Caerin 1 Peptides, the Potential Jack-of-All-Trades for the Multiple Antibiotic-Resistant Bacterial Infection Treatment and Cancer Immunotherapy

**DOI:** 10.1155/2022/7841219

**Published:** 2022-04-11

**Authors:** Liyin Xiao, Xiaodan Yang, Junjie Li, Pingping Zhang, Shuxian Tang, Dongmin Cao, Shu Chen, Hejie Li, Wei Zhang, Guoqiang Chen, Guoying Ni, Tianfang Wang, Xiaosong Liu

**Affiliations:** ^1^The First Affiliated Hospital of Guangdong Pharmaceutical University, Guangzhou, Guangdong 510080, China; ^2^Zhongao Biomedical Co. Ltd, Guangzhou, Guangdong 510080, China; ^3^Cancer Research Institute, First People's Hospital of Foshan, Foshan, Guangdong 528000, China; ^4^Genecology Research Centre, University of the Sunshine Coast, Maroochydore DC, QLD 4558, Australia

## Abstract

Antibiotic resistance-related bacterial infections and cancers become huge challenges in human health in the 21^st^ century. A number of naturally derived antimicrobial peptides possess multiple functions in host defense, including anti-infective and anticancer activities. One of which is known as the caerin 1 family peptides. The microbicidal properties of these peptides have been long discussed. The recent studies also established the usage of two members in this family, caerin 1.1 and caerin 1.9, in antimultiple antibiotic-resistant bacteria species. It is increasingly evident that caerin 1.1 and caerin 1.9 also contain additional activities in the suppression of tumor. In this review, we briefly outline the therapeutic potentials and possible mechanism of action of caerin 1.1 and 1.9 in the treatment of multiple antibiotic-resistant bacterial infection and cancer immunotherapy.

## 1. Introduction

Antibiotics, which have saved millions of lives from various infectious diseases as a meaningful medical discovery since the first prescription of penicillin in the 1940s, now confront a critical challenge at the beginning of the 21^st^ century with increasing amounts of antibiotic-resistant bacteria [1–4]. Due to the misuse and overuse of antibiotics, the emergence of antibiotic-resistant bacteria, especially those that exhibit multidrug resistance, called “superbugs,” courts the extensive uselessness of conventional antibiotics and therefore threatens the modern medical system [5–7]. The crisis of antibiotic-resistant bacterial infection existed as a global concern [2, 8]. By estimation, antibiotic-resistant bacteria result in approximately 25,000 deaths in Europe and 23,000 deaths in the United States, respectively, implying that the shortage of effective antibiotics poses an imminent threat to public health [6, 9, 10]. The global pandemics of antibiotic resistance greatly impact clinical practice in every field of health and medicine. There is an urgent and immediate need for new therapeutics with bactericidal activity against these multiple antibiotic-resistant bacteria.

Another worldwide disease that is highly weighed onto the global health burden is cancer, ranking as the first or second leading cause of death in most countries [11, 12]. The incidence of cancer is continuously rising, and it has been estimated that 19.3 million cases diagnosed with 10 million deaths worldwide in 2020, while a 45% increase is estimated to be 28.4 million by 2040 [11, 13]. Some cancers could be prevented or controlled through various measures, including tobacco control, vaccination, early detection, and promotion of healthy lifestyles. However, any administration is far from being ideal. In addition, patients suffering from chronic infections are more susceptible to cancer because of the impairment in the immune system, which hamper their abilities to fight invading pathogens [14]. The increase of inflammation becomes a possible factor in initiating cancer development [15]. Approximately 16% of cancer cases worldwide were attributed to certain infectious agents like bacteria, viruses, and parasites annually, ranging from 7% to 22% in less developed and more developed countries [16, 17]. At the same time, some cancers could develop to be more resistant to current therapeutic strategies. Therefore, the development of novel therapeutic strategies for pathogenic infections and malignant cancers remains a vital matter, especially for those with advanced and refractory diseases.

In recent years, a new promising class of molecules, naturally derived host-defense peptides (also known as antimicrobial peptides), has risen in attention. These molecules exert successful host defenses of eukaryotes against bacteria, protozoa, fungi, and viruses [18, 19]. Some of these biologically active compounds have emerged as promising therapeutic agents in some human diseases [20]. During the last three decades, hundreds of host-defense peptides have been isolated and identified from skin secretions from many species of Anura (frogs and toads) [21]. Caerin 1 peptides are one of the natural antimicrobial agents isolated from the skin secretions of Australian tree frogs of the genus *Litoria*. Many of these peptides exhibit antibacterial, antifungal, antiviral, antitumor, and immunomodulatory activities [22–25]. These findings provide the opportunity for the development of this class of specific and multifunctional antimicrobial agents as therapeutics against bacterial infection and cancer.

The aim of the present review is to offer a comprehensive and updated overview of studies addressing the therapeutic potential of caerin 1 peptides, mainly caerin 1.1 and 1.9, highlighting the opportunities offered by these compounds in the fight against pathogenic microbes, including antibiotic-resistant bacteria, and malignant tumors, but also the limits that may arise in their use for this type of application and future directions to optimize their biological activities.

## 2. The Structure of Caerin 1 Peptides Is Important for Their Action

**Figure 1:**
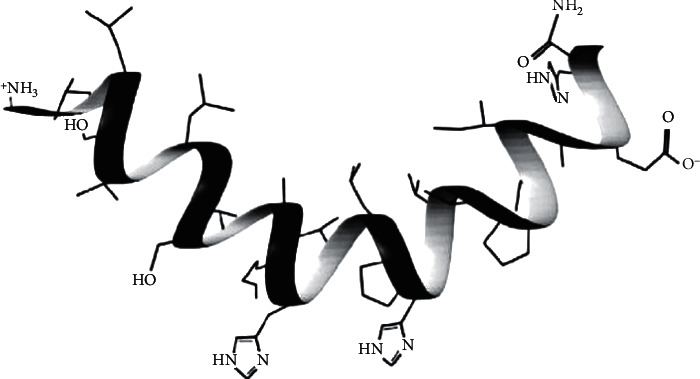
The 3D structure of caerin 1.1 adapted from reference [23].

Caerin 1 peptides consist of 25 residues. All members in this family share a similar primary structure with a consistent central fragment and a few amino acid variations in the sequence, which is critical for their defense activity. The general structure of caerin 1 peptides was identified based on caerin 1.1. Caerin 1.1 adopts an alpha-helical conformation which is separated by a flexible hinge region maintained by proline residues [22, 26]. The helix-hinge-helix structure allows the two helixes to freely orientate such that the side chains form hydrophobic and hydrophilic zones, resulting in an overall amphipathic molecule [27]. The structure of caerin 1.1 adopted from Bowie et al. is shown in Figure 1 [23]. The amphipathic characteristic appears critical for the effective interaction between caerin 1 peptides and lipid membrane. Another study showed that elimination of the proline-shaped central hinge structure of caerin 1.1 resulted in a conformational change of secondary structure and a loss of bactericidal activity [28]. In addition, all caerin 1 peptides share a common sequence and structural features in their N- and C- terminus, which has been linked to their antimicrobial activity [23, 26]. Additional amino acid tags to either end of caerin 1.1 induced changes in its bioactivity [29]. Therefore, both the amino acid composition and helix-hinge-helix structure are essential for the activities of caerin 1.1 and related peptides.

## 3. Caerin 1 Peptides “Kill” Antibiotic-Resistant Bacteria

### 3.1. Bacteria-Killing Activities

Caerin 1 peptides were initially discovered due to their activity in killing bacteria. Peptides in this family possess a wide-spectrum antibacterial activity toward a wide range of microbial targets with a low propensity for resistance development [22]. To date, more than 10 peptides were identified as members of caerin 1, and all showed some degrees of inhibitory effects against various bacteria. Each caerin 1 peptide displays its unique sequence, and the bacteria-killing ability of caerin against various bacterial pathogens *in vitro* has been well documented [26, 28, 30]. Caerin 1.1, the most representative active antimicrobial peptides in this family, was studied by many researchers, and we also found that caerin 1.9 showed a stronger antibacterial ability against some common bacterial pathogens. The minimum inhibitory concentrations (MIC, *μ*g/ml) of caerin 1.1 and caerin 1.9 against certain bacteria were verified by repeated broth microdilution method, and the results were organized in a summarized table (Table 1).

The explosion of antibiotic-resistant infections is increasingly prevalent in hospitals and communities, and these infections are resistant to traditional antibiotics [31, 32]. Among the hundreds of antibiotic-resistant pathogens found, Methicillin-resistant *Staphylococcus aureus* (MRSA) is the highly prevalent one, and it is related to massive infectious diseases [5]. Remarkably, our study found that the combination of caerin 1.1 and caerin 1.9 showed an additive effect against MRSA and *A. baumannii in vitro* and possessed bacterial suppression in murine skin MRSA infection for both Babl/c and C57BL/6 mice [30]. Although the bactericidal effect is generally more pronounced on Gram-positive bacteria, which is a common feature of most AMPs [22, 23], some Gram-negative bacteria, such as human pathogen *M. luteus* and aquaculture pathogen *P. anguilliseptica* and *V. anguillarum*, are sensitive to caerin 1.1 [26, 33]. Caerin 1.9 has stronger impacts on the inhibition of *S. aureus* and *S. hemolyticus*, as well as a drug-resistant bacteria strain, MRSA, than caerin 1.1 [30]. However, caerin 1.9 contains a weaker antibacterial effect upon *S. epidermidis*, *S. uberis*, and *P. multocida* [26].

In order to evaluate the bacteria-killing efficacy of caerin peptides, we also performed broth microdilution assays to compare the MIC values between several common antibiotics with caerin 1.1 and 1.9 (unpublished data). Polymyxin B is a traditional antibiotic managing Gram-negative bacterial infections. The MIC results demonstrated that either caerin 1.1 or caerin 1.9 displayed stronger antibacterial effects than polymyxin B against Gram-positive bacteria, MRSA, *S. aureus*, and *S. hemolyticus*, but weaker abilities in killing Gram-negative bacteria, *E.coli* and *P. aeruginosa*. Sodium fusidate and dalbavancin possess effective bactericidal activity against most Gram-positive bacteria. They had an advantage over caerin peptides in killing MRSA and *S. aureus*, whereas caerin 1.1 and caerin 1.9 had a better performance than sodium fusidate, but not dalbavancin, in inhibiting *S. hemolyticus*. Different from these antibiotics that contain restrictions in killing limited bacteria, caerin 1 peptides possess wide-spectrum antibacterial activity. The killing efficacy varied regarding the unique characteristics of each bacterium.

The low tendency to develop resistance makes AMPs alternative drugs, exhibiting strong and rapid antibacterial activity either alone or in combination with antibiotics. It has been also evidenced that AMPs and existing antimicrobial agents may be used in combination to better combat bacterial pathogens, especially antibiotic-resistant bacteria [34, 35]. The ability of AMPs to permeabilize bacterial membranes plays a role in their synergized effects with the combination of antibiotics [36]. Therefore, the combined application of caerin 1 peptides with antibiotics may be a promising strategy to improve the bacteria-killing efficacy while reducing the propensity of pathogens to develop antibiotic resistance. The potential usage of caerin-combined therapeutics for combating bacteria points to the importance of further investigation of these effects.

### 3.2. Membrane Action of the Caerin 1 Mediated Cell-Killing Activity

The multihit mechanism of the membrane actions makes contributions for AMPs to evade the development of resistance [22], which is also suggestive in caerin 1 peptides. Membrane perturbation becomes a reliable explanation for the leading mechanism of their bactericidal activity. Other than traditional antibiotics that are typically directed at structural or enzymatic targets that are unique to certain bacteria, many antimicrobial peptides, including caerin 1 peptides, kill bacteria depending on their ability to interact with bacterial membranes or cell walls [37–39]. This membrane-active activity is associated with the structural properties of the peptide itself, with features including length, sequence, charge, the formation of helical structure, hydrophobicity, and amphiphilicity [40]. In addition, the concentration of peptides absorbed by the membrane surface and the membrane environment, such as the envelope of cells and the composition of the lipid bilayer, influences the interaction with membrane lipids [41].

Generally, AMPs exhibit a net positive charge and a high ratio of hydrophobic amino acids, allowing them selectively to bind to negatively charged lipid membranes [42]. The electrostatic interaction between cationic peptides and negatively charged phospholipids led to the disorder of lipid chains and destroyed membrane integrity, promoting lysis of the targeted microbes [43–45]. Caerin 1.1 has been shown to destabilize the bacterial membrane through direct insertion as a carpet-like shape at sufficiently high peptide concentrations, or alternatively, the transmembrane alpha-helices may also oligomerize into either “barrel-stave” or “toroidal pores” [37, 38, 41, 46]. They display effective antibacterial activity through multiple membrane actions.

The carpet mechanism is initiated as peptides assemble in alpha-helical form with their hydrophilic axis parallel to the membrane surface by interacting with lipid head groups [28]. The hydrophobic helix penetrates the bilayer to interact with the lipid acyl chain interior, forming a carpet-like pattern. Above a critical concentration, transient holes are formed due to surface tension on the bilayer, and the membrane degrades into micelle-like complexes, mediating cell death [47].

In the “barrel-stave mechanism,” electrostatic attraction drives the peptides to insert perpendicularly into the bilayer, while recruitment of additional peptides to align the interior of the bilayer subsequently results in a peptide-lined hydrophilic transmembrane pore. In contrast, the formation of “toroidal pores” is due to the force of oligomerization that alters acyl chain orders to induce the formation of membrane curvature such that the inner and outer surfaces become continuous, resulting in a pore lined by both peptides and the head groups of the phospholipids [48].

The formation of either carpet structures or transmembrane pores impairs normal membrane function. The pores act as nonselective channels that allow an excessive flow of ions and molecules, thus disrupting the imbalance of homeostasis and eventually leading to cell lysis. It has been also demonstrated that AMPs possess immune responses to mediate bacteria clearance [49, 50]. Another *α*-helical antimicrobial, sublancin, modulated the innate immune system by activating phagocytosis of macrophages and increased T cell activation [51]. Similar or distinct immune activities to defense microbes might present in the process of anti-infection of caerin 1.1 and 1.9.

Translocation of caerin 1 peptides via membrane permeabilization might also be an important factor for the bacteria-killing activity in the cytoplasm, where they can target cellular processes. Some AMPs have been proved to alter DNA/RNA and protein synthesis, protein folding, enzymatic activity, and cell wall synthesis [42]. Although there is a lack of studies of the site of action and intracellular targets of caerin 1 peptides, a mass of evidence showed that some peptides are more likely to target and destroy certain kinds of bacteria membranes depending on the features of membranes. The binding selectivity of caerin 1.1 and caerin 1.9 is due to differences in the membrane composition in distinct microbes. Caerin 1 peptides possess cationic properties, and the presence of positively charged residues could be an important contributor to their selectivity [27, 37]. They possess a higher preferential selection in targeting negatively charged bacteria membrane [23, 26]. Despite the strong antimicrobial activity in killing bacteria, caerin 1 peptides do not cause damage to normal eukaryotic cells that are mainly composed of neutral lipids, providing them potential use in infectious diseases [52]. Multiple studies have also discussed that the bacterial recognition of caerin 1 peptides was based on the cationic contents and the membrane states: in the in vitro membrane mimetic environment, the peptides were promoted to form an alpha-helical secondary structure, and the interaction of anionic phospholipid was more pronounced than that of positively charged bilayer or neutral bilayer [37, 38, 40, 48]. Solid-state NMR studies also revealed that the molecules of caerin 1.1 do not penetrate deeply into neutral or positively charged membranes [28]. In addition, higher content of cholesterols in mammalian membranes hampers AMPs to disrupt lipid bilayer structures [53]. Therefore, the cationic antimicrobials are selectively toxic to bacteria rather than eukaryotic cells via electrostatic interactions with negatively charged membrane lipids during the process of bacteria clearance.

Other than the special classification between normal mammalian cells and bacteria cells, caerin 1.1 and related peptides generally possess a lower inhibitory effect on Gram-negative bacteria that obtained a more complex protective structure than on Gram-positive bacteria. The outer membrane of Gram-negative bacteria is negatively charged because of anionic lipopolysaccharides (LPS). The cationic antimicrobial peptides bind with anionic lipopolysaccharides; thus, a higher concentration of peptides is required for membrane interaction [45]. Instead, caerin 1.1 killed *B. subtilis* (a Gram-positive bacteria without outer membrane protection) with low peptide concentration since the presence of negatively charged teichoic and lipoteichoic acids in the peptidoglycan on the membrane surface might induce peptide attraction [41]. This evidence indicated that the membrane composition plays a crucial role in caerin 1.1 peptide incorporation, depending on the distinctive features of different bacteria.

## 4. Caerin 1.1/1.9 against Other Infectious Pathogens

Besides the extensive studies that have confirmed the effective activity in bactericidal, the inhibitory ability of caerin 1 peptides was reported in viruses and fungus. Caerin 1 family peptides possessed anti-infective effects as they inactivated HIV with limited toxicity to normal mammalian cells [25]. Caerin 1.1 and caerin 1.9 were reported to efficiently inhibit HIV infection of T cells at concentrations nontoxic to targeted T cells and normal cells, and these peptides were able to hamper the transmission of the virus from dendritic cells to T cells by killing HIV captured by DCs [54]. In a recent study, caerin 1.9 enabled to inhibit the growth of *Neisseria* (a sexually transmissible pathogenic microorganism) and HIV while maintaining less harmful effects to protective lactobacilli (a member of the vaginal microbiome), suggesting its potential candidate to protect against gonorrhea [55]. A recent *in silico* study finds that caerin 1.6 and caerin 1.10 highly interacted with Arg995 located in the S2 subunits of spike surface viral protein (Spg), which is the key subunit essentially needed in viral fusion and entry into the host cell through the angiotensin-converting enzyme 2 of SARS-CoV-2 (ACE2) [56]. This high affinity might reduce the interaction between Sgp and the ACE2 receptor, suggesting a therapeutic method for SARS-CoV-2 infection, though an experimental validation needs to be performed.

The inactivation of viral pathogens was mediated by directly attacking the viral envelope upon exposure, followed by the release of viral core protein, to avoid the infection of the target cells, and the disruption of the virion membrane was virus-specific and independent of envelope glycoproteins [25, 54, 57]. This mechanism shares the similarity to interfering bacterial membrane: the outer membrane is impaired by interaction with these cationic antimicrobial peptides. The effectiveness of the peptides to destroy the viral envelope is highly correlated with the ability to inhibit infection. In addition to the capability of the caerin 1 peptides to directly bind viral envelopes, it is widely accepted that they may also contain other immunomodulatory activities, which can enhance their antivirus responses. Several other amphibian AMPs have been shown to affect lymphocyte activation and cytokine production [58–61]. Whereas few studies addressed that the immune response might be induced by caerin regarding viral inhibition, more research is required to evaluate their suppressing or enhancing effects as well as immunomodulatory effects on the immune system [62].

Several members in the caerin 1 family appear to be safe and promising microbicides to limit HIV transmission or pathogenic vaginal bacteria. However, the interplay of the vaginal microbiome and introduced viruses is complicated. Additional research is needed to determine whether and to what extent the introduction of antimicrobial peptides such as caerin 1 peptides would be beneficial.

## 5. Caerin 1.1 and 1.9 in Cancer Therapy

### 5.1. Caerin 1.1 and Caerin 1.9 Inhibit Cancer Cell Growth Both In Vitro and In Vivo

Although numerous chemotherapeutic drugs have been successfully developed for the treatment of cancers, severe side effects and usage limitations are prevalent as they target all rapidly dividing cells, rather than solely cancerous cells. The aggressive cancer therapies might further weaken the patients' ability to fight infectious agents [14, 63]. At the same time, tumor cells could develop multiple resistance to the current therapeutic strategy, making the cancer therapy ineffective [64–66]. Therefore, the development of novel antitumor molecules for malignant cancers remains a vital matter, especially for those with advanced and refractory diseases.

Recently, several cationic antimicrobial peptides which display antitumor activity have received attention as alternative agents to overcome the limits of current cancer chemotherapy [67–69]. These peptides are selective cytotoxicity for cancer cells, even multidrug-resistant cancer cell lines, but not normal mammalian cells with a low propensity for resistance development [70, 71]. Caerin 1 peptides containing similar structure and membrane-interacting action were also suggested to exert anticancer properties [22, 37]. Caerin 1.1 and 1.9 peptides significantly inhibit the proliferation of several different cancer cells, such as cervical cancer cell line TC-1 [72] and HeLa [73, 74], breast cancer cell line MCF-7 and Skbr-3, thyroid cancer cell line B-CPAP and CAL-62, and melanoma cell line B16 (unpublished data) *in vitro*, and an additive effect was observed when using in combination [75, 76]. Confocal microscopic images revealed that both caerin 1.1 and caerin 1.9 penetrated the HeLa cell membrane and accumulated primarily in the nuclei with different kinetics. The peptide internalization was observed in minutes and enhanced with time, and there was a higher cell uptake of caerin 1.9 than caerin 1.1 in HeLa cells [74]. Neither caerin 1.1 nor caerin 1.9 affected the proliferation of the normal epithelial transformed cells, NP-69, at similar concentrations that inhibited the growth of cancerous cells [72].

Due to the tumor-specific targeting ability, caerin 1.1 and 1.9 have been studied for their potential use for diagnostic imaging and radiotherapy in oncology. Administration of ^131^I-caerin 1.1 to thyroid cancer-bearing mice resulted in the inhibition on malignant cell viability, the increase of iodine uptake, and the reduction of tumor mass, suggesting that radiolabeling caerin peptide may become a theragnostic tool for radioiodine refractory thyroid cancer [75, 77]. With the antitumor immune effect activated by caerin 1.1 and 1.9, synergistic strengthening of tumor-killing ability was observed with the combination of radiotherapy to enhance the efficacy of cancer therapy. Similarly, caerin 1.9 labeled with ^125^I improved the inhibition of cell viability of MCF-7 breast cancer cells *in vitro*, and the radioiodine peptides tend to accumulate at tumor tissue *in vivo* [76]. Also, on the basis of much higher radioiodine delivery through caerin labeling, it allowed the reduction of the delivery of radiation doses to the patients to attain high efficiency to the tumor loci. Moreover, caerin 1.1 and 1.9 inhibited TC-1 [73] and B16 (unpublished data) tumor growth *in vivo* when injected intratumorally, and the inhibition requires an intact adaptive immune system and the existence of T cells [78].

### 5.2. Cancer Cell-Killing Mechanism

Caerin 1 peptides exert cytolytic activity against cancer cells through introducing ion-permeable pores on the cell membrane, which is similar to what happens on bacteria cells [22]. The differences between normal mammalian cells and malignant cancer cells in membrane composition contributed to the target selectivity of caerin 1 peptides. One of the direct determinants is the charge difference introduced by the alteration of lipid composition. The membrane of cancer cells expresses a high level of negatively charged gangliosides [79–81], while the membrane of normal cell membranes consists largely of neutral phospholipids that are less attractive [37]. It has been proposed that the membrane destruction that results in leakage of intracellular contents contributes to the inhibition of cancer cells by caerin 1.1/1.9 activity [76].

However, some researchers have realized that cationic antimicrobial peptides do not only function in membrane disruption; the immune system also plays a role in caerin peptide-mediated bioactivity [82, 83]. The immunomodulatory effect of these peptides plays a role in interacting with the host cells by influencing diverse signaling cascades [71]. For example, *β*-defensins, a kind of small, cationic, host-derived AMP, act as a ligand for the CCR6 and CCR2 chemokine receptors to induce the chemotactic activity of lymphocytes [84]. Caerin 1.1/1.9-treated TC-1 cells secrete proinflammatory cytokines, such as TNF-*α*, IL-1*β*, IL-6, and MCP-1, that promote immune cell trafficking to the tumor site [72]. Caerin 1.1/1.9-mediated death requires an intact adaptive immune system: the tumor-killing effect disappeared when the adaptive immune system is absent [78]. The activation of the TNF-*α* signaling pathway, an apoptotic signal, was reported to be important for the cancer-killing ability of caerin 1.1 and 1.9 [74], which was suggested to be regulated by Stat1 [85]. The TMT-labeling proteomics showed a significant difference in protein expressions in HeLa cells and cell growth environment 24 hr post caerin treatment, including the biological processes of translation, apoptosis, glycolytic metabolism, and protein folding. Meanwhile, the recruitment of T cells to the cell growth environment was promoted by the activation of the TCR pathway, suggesting that the peptide-stimulated HeLa cells were highly sensitive to T cell-mediated killing [74].

Moreover, the intracellular target for caerin 1 peptides may exit. Once they approach the nucleus, they may exert other biological functions by interacting with some proteins to impact the cellular functions. Indeed, we demonstrated that either caerin 1.1 or 1.9 could penetrate into the cytoplasm of Hela cells and distribute on the nucleus membrane, entering with different velocities [74]. Ongoing studies are required to investigate the potential mechanism of caerin other than membrane penetrating.

### 5.3. Caerin 1.1/1.9 Drastically Improved the Survival Time of Anti-PD-1-Treated and Vaccinated TC-1-Bearing Mice

Current vaccines are extremely effective at preventing viral infection; however, they are merely prophylactic and fail to clear established infections. HPV-related cancers are typically treated with multimodal therapy, including surgery, chemotherapy, and radiation. Immunotherapy has represented a breakthrough in recent years, with confirmed therapeutic responses reported with immune-checkpoint blockades (ICB), such as PD-1 and CTLA-4 blocking monoclonal antibodies, in a variety of tumor types [86, 87]. Transfer of CAR-T cells obtained significant efficacies for lymphoblastic leukemia, and pembrolizumab (PD-1) has been approved by FDA in the treatment of lung cancer [88–91]. Active immunization showed some kinds of benefit to precancer conditions, such as CIN or VIN for HPV-related malignancies [92–95]. However, only a fraction of patients with so-called “hot” solid tumors respond partially to the ICB, CAR-T, or therapeutic vaccination therapy [96–98]. Therefore, increasing the efficacy of cancer immunotherapy remains a challenging task for clinicians and scientists.

Many strategies have been investigated for the development of therapeutic vaccines. [99, 100]. Therapeutic vaccines incorporating a cytokine, an interleukin-10 signaling inhibitor, drastically increase vaccine-induced antigen-specific T cell responses, attracting more T cells to the tumor site, and furthermore prolong the survival time of tumor-bearing mice [101, 102]. The effect of an IL-10 inhibitor regulated the PI3K/AKT signaling pathways to promote the antigen-specific CD8^+^ T cell responses and the alteration of the biological function of tumor-infiltrating macrophages [103]. The immunomodulating ability of caerin 1 peptides makes them become another candidate to inhibit tumor cell proliferation by improving the immune-suppressive tumor microenvironment. These peptides were able to increase the survival time of TC-1 tumor-bearing mice after therapeutic vaccination with an HPV16E7 peptide-based vaccine containing IL-10 inhibitor via increased recruitment of T cells to the tumor site, probably by TC-1-promoted secretion of proinflammatory IL-6 [78]. Moreover, PD-1 inhibitor combined with therapeutic vaccine synergistically suppressed the growth of tumor in an HPV16 E6/7-transformed TC-1 murine tumor model [104]. Remarkably, intratumorally injection of caerin 1.1 and caerin 1.9 in conjunction with an HPV16 E7 peptide-based vaccine containing IL-10 inhibitor and PD-1 blockade in tumor-bearing mice significantly suppresses tumor growth and prolongs their survival time [85]. The average survival time between the mice-immunized therapeutic vaccination in the combination of IL-10 inhibitor and PD-1 with or without caerin 1.1 and 1.9 was 37 days vs. 16 days. The expression of memory CD8^+^ T cells and effector-memory CD8^+^ T cells was further enhanced with additional caerin treatment. Recently, similar results were observed in a murine B16 tumor model, suggesting that caerin 1.1 and 1.9 were able to improve the tumor immune-suppressive environment in both HPV+ and HPV- tumors (unpublished data). Therefore, caerin1.1/1.9 is better than the current ICB therapy at improving the survival of tumor-bearing mice if they are vaccinated with the therapeutic vaccine. The possible mechanisms might result from the better interruption of the tumor microenvironment of caerin 1.1/1.9, as they can activate T cells, NK cells, macrophages, and dendritic cells [85].

Single-cell RNA sequencing analysis also revealed that caerin 1.1 and caerin 1.9 upregulated the populations of immune-activating macrophages (M1 phenotype) and natural killer cells while dramatically reducing the number of immunosuppressive macrophages (M2 phenotype) and protumorigenic B cells [85]. The macrophages from caerin-treated mice secreted a high level of IL-12 and low levels of IL-10 and IL-6 (unpublished data). Additionally, injection of caerin largely induced *MHCII^high^* and *Ear2^high^* macrophages in tumors. Further analysis revealed that the IFN-*α* signaling pathway was activated in tumor-infiltrating macrophages.

Taken together, caerin 1.1 and 1.9 treatment enabled to improve antitumor responses probably via the modulation proinflammatory apoptosis of tumor cells, resulting in the more local release of proinflammatory cytokines, therefore alleviating the tumor immune-suppressive environment. The tumor-infiltrating T cells were more activated following the administration of caerin 1.1 and 1.9 through direct or indirect interaction with tumor-infiltrating macrophages. Finally, the tumor cells became more sensitive to T cell-mediated killing.

### 5.4. Caerin 1 Peptides Are Well-Tolerated in Rats and Stable in Environment

To better develop the use of caerin 1 peptides for cancer therapy, the pharmacokinetics profile, the tissue distribution report, and the acute safety study of the representative caerin 1.9 peptides in SD rats were studied (Yang et al., submitted). The results showed that subcutaneous injection of caerin 1.9 is considered safe at a dose up to 100 mg/kg, without leading to recipient rat death or any remarkable organ dysfunction. The plasma concentration of caerin 1.9 reached to peak at 1 hour after a single administration and degraded to the basal level in hours. As injected concentration increased, the T1/2 was prolonged, and the Cmax, the AUC0-last, and the homeostasis volume in vivo were elevated. No accumulation of caerin 1.9 in plasma was detected after repeated subcutaneous injection of 10.0 mg/kg for 14 days. The assessment of pharmacokinetics and tissue distribution suggested that caerin 1.9 is well tolerated in rats.

Furthermore, caerin 1.1 and 1.9 were active at inhibiting TC-1 or HeLa cell growth at pH 5.5-7.4 [78] and active at inhibiting MRSA growth *in vitro* between pH 5-11 [30]. After heating at 100°C for 10 minutes, they maintained their ability to inhibit TC-1 cell growth *in vitro*. And caerin 1.1 remains similar antiproliferative activity against TC-1 cells *in vitro* when stored at room temperature for 14 months. Caerin 1.9 was similarly active at 11 months (unpublished data), but it showed reduced bioactivity at 14 months. The stability test provided the basis for the development and the utilization of caerin 1.1/1.9 as potential therapeutics.

## 6. Concluding Remarks and Future Perspectives

Caerin 1 peptides are emerging as novel alternative therapeutic molecules upon multiple diseases such as bacterial infections, viral infections, and cancers. Caerin 1.1 and 1.9, originally isolated the skin secretion from Australian tree frog, have broad-spectrum antibacterial activity and are able to inhibit multi-antibiotic-resistant bacteria growth both *in vitro* and *in vivo*. Unlike antibiotics, they do not induce resistant strains when cultured *in vitro* for 30 rounds [30]. Caerin 1 peptides also mediate tumor cell apoptosis, probably through the TNF-*α* signaling pathway, and increase the efficacy of ICB and therapeutic vaccination therapy. Furthermore, caerin 1 peptides, when intratumorally injected, drastically improve the PD-1 blockade and therapeutic vaccinated tumor-bearing mice by modulating the tumor immune-suppressive environment by changing the heterogenicity of tumor-infiltrating macrophages. Importantly, caerin 1 peptides are environmentally stable and maintained their bioactivity at room temperature for over 1 year (unpublished data), with a wide pH range. And caerin 1.9 is well tolerated in rats when subcutaneously injected at a dose of 100 mg/kg. Caerin 1 peptides, therefore, are a promising candidate for the management of multi-antibiotic-resistant bacterial infection and cancer immunotherapy. In the future, combination strategies involving this novel therapeutic peptide with conventional cancer therapies may improve treatment outcomes.

However, important issues are required for further attention for better understanding the biological function and optimizing the bioactivity of caerin peptides. For example, although membrane interaction with bacterial and cancerous cells is the key determinant for caerin-mediated bacterial and cancerous cell death, how caerin peptides interact with bacteria and cancer cell membrane remains elusive. Proteomic analysis suggested that caerin 1 peptides induced cancer cell apoptosis through the TNF-*α* signaling-mediated pathway and inhibit the EGFR signaling pathway; however, currently, it is not clear which protein or proteins in these pathways interact with caerin 1 peptides and whether through direct or indirect interaction, or at transcription, translation, or through epigenetic mechanisms. Caerin 1 peptides increase the efficacy of therapeutic vaccination and ICB therapy but drastically improve the effects when ICB and therapeutic vaccination combined therapy in tumor-bearing mice, by further modulating the function of tumor-infiltrating macrophages. Clearly, ICB and therapeutic vaccination provided an ideal tumor microenvironment that is suitable for caerin 1 peptides to execute their effort, which in turn significantly prolonged the survival time of the tumor-bearing mice. Therefore, it is of great interest to investigate the difference of the tumor microenvironment, especially tumor-infiltrating cell functions, and the functional changes of the tumor-infiltrating macrophages by new techniques such as multiple omics and single-cell RNA sequencing, including spatial transcriptome techniques, combined with new flow cytometry technologies. Finally, peptides usually have short blood half lift and can easily be digested by protease in peripheral blood and tissues. Increasing the half-life and optimizing their delivery in the human body, probably through incorporation with nanoparticles or liposomes for controlled release, are needed for them to be used in clinics.

## Figures and Tables

**(a):** 

Name	Sequence
Caerin 1.1	GLLSVLGSVAKHVLPHVVPVIAEHL-NH_2_
Caerin 1.9	GLLSVLGSVAKHVLPHVVPVIAEHL-NH_2_

**(b):** 

MIC (*μ*g/ml)		
	Caerin 1.1	Caerin 1.9
Gram + bacteria strains		
MRSA	15	3.75
*Staphylococcus aureus*	15	3.75
*Streptococcus hemolyticus*	15	7.5
Gram – bacteria strains		
*Acinetobacter baumannii*	15	7.5
*Escherichia coli*	60	30
*Pseudomonas aeruginosa*	120	60

Each result is the representative of two independent experiments.
